# Association between acromegaly and a single nucleotide polymorphism (rs2854744) in the IGFBP3 gene

**DOI:** 10.1186/s12881-018-0698-2

**Published:** 2018-10-05

**Authors:** Ming Gao, Bin Zhu, Zhe Xu, Shujun Liu, Jiajia Liu, Guojun Zhang, Yang Gao, Yubo Fan, Xixiong Kang

**Affiliations:** 1 Laboratory Diagnosis Center, Beijing Tiantan Hospital, Capital Medical University, Beijing, 100050 China; 2 Beijing Engineering Research Center of Immunological Reagents and Clinical Research, Beijing, 100050 China; 3 Department of Pharmacy, Beijing Tiantan Hospital, Capital Medical University, Beijing, 100050 China; 4 Monogenic Disease Research Center for Neurological Disorders, Beijing Tiantan Hospital, Beijing, 100050 China; 5 Lab of Biological Science and Medical Engineering, Beihang University, Beijing, 100191 China

**Keywords:** Insulin-like growth factor binding protein-3, Single nucleotide polymorphism, Acromegaly, Pituitary adenoma, Case-control study

## Abstract

**Background:**

It has been reported that the single nucleotide polymorphism (SNP) rs2854744 at the − 202 locus of insulin-like growth factor binding protein-3 (IGFBP3) is associated with serum levels and a number of malignancies. However, the effect of IGFBP3 gene polymorphism on acromegaly is less clear. Therefore, in the current study, we aimed to investigate whether the −202A/C polymorphism of IGFBP3 constitutes a risk factor for acromegaly.

**Methods:**

The study included 102 acromegalic patients and 143 control subjects in Beijing Tiantan Hospital. The genotyping of IGFBP3 was carried out using the MassARRAY method. Serum IGFBP3 concentrations were also determined. Odds ratios (ORs) and 95% confidence intervals (CIs) were used to evaluate the associations of genetic polymorphisms with the development of acromegaly and its different subtypes.

**Results:**

The study revealed that the C allele of rs2854744 was associated with a reduced risk of acromegaly (OR 0.594, 95% CI 0.388–0.909), as well as with the female (OR 0.385, 95% CI 0.206–0.72), macroadenoma (OR 0.557, 95% CI 0.347–0.893) and monotherapy (OR 0.512, 95% CI 0.316–0.828) subgroups under the additive model. A higher serum IGFBP3 level was observed in patients with the AA genotype, but this difference was not significant (*P* = 0.331).

**Conclusion:**

This study is one of the first to show that the IGFBP3 polymorphism may have an influence on serum levels and that the C allele of rs2854744 is associated with a reduced risk of acromegaly. This correlation was more prominent in females, those with large tumours and those treated with monotherapy in a Chinese population. Genetic polymorphism of IGFBP3 may be involved in the development of acromegaly.

## Background

With a prevalence of 14–22%, pituitary adenoma is one of the most common intracranial neoplasms. An increasing number of pituitary surgeries are performed in our hospital. Somatotroph adenomas, which constitute the commonly diagnosed secreting adenomas (10–15%), are the most frequent causes of acromegaly [1]. However, acromegaly is a relatively rare disease. It is attributable to excessive levels of growth hormone (GH) and secondary elevation of insulin-like growth factor 1 (IGF-1) levels, which lead to somatic overgrowth, multiple comorbidities and an increased risk of death [2].

Excessive secretion of GH causes an overproduction of IGF-1 in the liver and other systemic tissues. A majority of circulating IGF-1 is bound to IGF-specific binding proteins (IGFBPs). There are six types of IGFBPs (IGFBP1~ IGFBP6). IGFBP3 is the major subtype that transports more than 75% of serum IGF. IGFBP3 modulates the bioavailability of IGF by increasing its half-life and modifying its biological activities on target tissues [3]. IGFBP3 not only inhibits the effect of IGF-1 by blocking the binding of IGF-1 to its receptor but also enhances the action of IGF-1 and, consequently, stimulates cell growth by protecting IGF-1 from degradation under some conditions [4]. The determination of IGFBP3 circulating levels and gene polymorphisms is useful for the assessment of growth disorders and various kinds of cancer [5–8].

The SNP rs2854744 at the − 202 site of the IGFBP3 gene, which is located at chromosome 7p, has been reported to be correlated with the circulating levels of IGFBP3 in both in vitro and in vivo studies [9]. Several studies have shown an association between the IGFBP3–202 A/C polymorphism and both the clinical features and therapeutic responses of acromegaly patients [10–12]. To our knowledge, there has been only one small-scale study that reported a difference in the genotype-specific distribution of IGFBP3 between acromegaly patients and controls. However, they did not study the association of the SNP with acromegaly susceptibility [10]. Pituitary adenomas can be classified into various subtypes according to multiple clinical and imaging characteristics. Therefore, we aimed to examine the correlation of IGFBP3 genetic polymorphisms at the -202 site with the susceptibility to acromegaly and its different subtypes in the Han Chinese population.

## Methods

### Subjects

This was a case-control study. Overall, 102 acromegaly patients (56 men, 46 women, mean age 42.2 ± 10.24 years) were consecutively recruited from June to December in 2017 among inpatients in Beijing Tiantan Hospital. The diagnosis of acromegaly was based on the Endocrine Society Clinical Practice Guidelines [13]: (1) typical acromegaly clinical manifestations; (2) serum nadir GH concentration > 1 ng/mL after a 75 g oral glucose tolerance test (OGTT) or fasting GH value > 2.5 ng/mL; (3) serum IGF-1 levels above the normal age-adjusted range; and (4) enhanced magnetic resonance imaging (MRI) showing a pituitary tumour in the sellar area. In addition, every patient underwent surgery, and the presence of a pituitary adenoma was confirmed by postoperative pathological examination.

The control group was enrolled 143 individuals who were matched by age and sex with the acromegaly group. Most of the control individuals were seemingly healthy individuals, and the minority was craniocerebral trauma inpatients. Baseline data and medical histories were recorded. The exclusion criteria for the controls were as follows: (1) having a history of cancer or another serious chronic disease; (2) having a familial history of pituitary disease; and (3) not providing consent for genetic studies. All patients and controls were informed about the purpose of the protocol and signed consent forms. The protocol was approved by the Ethics Committee of Beijing Tiantan Hospital.

### Data collection and classification

Detailed clinical and imaging data were acquired upon admission for all participants. None of them had a familial history of pituitary diseases or acromegaly-related clinical syndromes. According to MRI reports, the adenomas were classified as either a microadenoma (larger diameter ≤ 10 mm) or a macroadenoma (larger diameter > 10 mm). Since some acromegaly patients had previously undergone pituitary surgery, the remainder of the patients were followed up at 4~ 10 months by observation of hospitalization for repeat surgery, medical treatment or radiotherapy. All patients were divided into two groups: a monotherapy group (patients only had one surgical treatment) and a combination therapy group (Table 1).

**Table 1:** Demographic characteristics of acromegaly patients and controls

Variables	Total	Case N (%)	Control N (%)	P^a^
N	245	102	143	
Age	42.21 ± 11.05	42.22 ± 10.24	42.2 ± 11.63	0.993
Gender				
Male	126(51.4)	56(54.9)	70(49)	0.358
Female	119(48.6)	46(45.1)	73(51)	
Smoking status^b^				
Ever	50(22.1)	28(27.5%)	22(17.7%)	0.08
Never	176(77.9)	74(72.5%)	102(82.3%)	
Drinking status^b^				
Ever	47(20.8)	26(25.5%)	21(16.9%)	0.115
Never	179(79.2)	76(74.5%)	103(83.1%)	
Tumour size^c^				
Microadenoma		13(14.1)		
Macroadenoma		79(85.9)		
Treatment method				
Combination therapy		24(23.5)		
Monotherapy		78(76.5)		

^a^T test was used for age, and a χ2 test was used for other binary variables. ^b^Data for 124 subjects in the control group were available; ^c^Data for 92 subjects in the case group were available

### DNA isolation and genotyping

Blood samples from each participant were collected in tubes containing ethylene diamine tetraacetic acid (EDTA) and stored at − 80 °C until use. Genomic DNA was extracted from peripheral white blood cells using a Qiagen DNA purification kit (Qiagen, Hilden, Germany). The DNA quantity was determined using a NanoDrop 2000 spectrophotometer (Thermo Fisher, Waltham, MA, USA). Polymerase chain reaction (PCR) primer pairs were used to amplify rs2854744 in IGFBP3: GGTTCTTGTAGACGACAAGG (forward primer) and GTGCAGCTCGAGACTCGCC (reverse primer). Genotyping analysis of the included population was performed using time-of-flight mass spectrometry on a MassARRAY iPLEX platform (Sequenom, San Diego, CA, USA) in Bio Miao Biological Technology (Beijing).

### Determination of serum IGFBP3 concentration

The blood samples from the patients were collected in the fasting state. IGFBP-3 levels were determined by chemiluminescent quantitative measurement using IMMULITE 2000 IGFBP-3 kits (Siemens Healthcare Diagnostics, United Kingdom) on the IMMULITE 2000 analyser (Siemens Healthcare Diagnostics, USA). The analytical sensitivity for IGFBP-3 was 0.1 μg/mL.

### Statistical analysis

Patient characteristics are expressed as the mean ± standard deviation (SD) for continuous variables, whereas categorical variables are presented as an absolute count and a percentage. A Kolmogorov-Smirnov test was used to test the assumption of normality. When a distribution was normal, Student’s t test was used for the assessment of statistically significant differences. Hardy-Weinberg equilibrium (HWE) was assessed by a χ^2^ test, and *P* > 0.05 indicated equilibrium. The differences in genotypic and allelic frequencies between groups were examined by Pearson’s chi-square test or Fisher’s exact test. ORs and 95% CIs were calculated to assess the associations of IGFBP3 -202A/C genetic polymorphisms with the development of acromegaly under dominant, recessive and additive genetic models. Logistic regression analysis was used to evaluate the contribution of genetic and non-genetic factors to the development of acromegaly (adjusted for age, sex, and smoking and drinking habits). Two-tailed *P* < 0.05 indicated statistical significance. All statistical analyses were conducted with SPSS version 20.0 (SPSS Inc., Chicago, USA).

## Results

### General characteristics of subjects

The demographic data of 102 acromegalic patients and 143 controls are summarized in Table 1. There was not a significant difference between the acromegalic cases and the controls in age, sex, and smoking and drinking status (*P* = 0.993, 0.358, 0.08 and 0.115, respectively). In the acromegaly group, 14.1% of patients were diagnosed with a microadenoma, and 23.5% of patients needed combination therapy. The genotyping call rate was more than 98% for IGFBP3 rs2854744. The observed genotype distribution for rs2854744 was in HWE in the controls (χ2 = 0.38, P_HWE_ = 0.236). The minor allele frequency (MAF) of rs2854744 was 0.262 (Table 2).

**Table 2:** IGFBP3 rs2854744 genotype distribution in acromegaly patients and controls

Genotype	Case [N(%)]^a^	Control [N(%)]^b^	MAF	P_HWE_	OR (95% CI)	P
AA	63(63)	72(50.7)	0.262	0.236	1	
AC	33(33)	54(38)			0.698(0.403–1.21)	0.2
CC	4(4)	16(11.3)			**0.286(0.091–0.899)**	**0.032**
A allele	159(79.5)	198(69.7)			1	
C allele	41(20.5)	86(30.3)			**0.594(0.388–0.909)**	**0.016**

^a^Genotypic data for 100 subjects in the case group were available. ^b^Genotypic data for 142 subjects in the control group were available. *MAF* minor allele frequency, *HWE* Hardy-Weinberg equilibrium. Significant associations are marked in bold

### Associations of rs2854744 with the risk of acromegaly

The genotype distribution of IGFBP3 rs2854744 between the acromegalic case group and the control group is shown in Table 2. The AA genotype was the most prevalent, followed by the AC genotype, and the CC genotype was the least prevalent. We found that the C allele was associated with a decreased risk of acromegaly compared to the A allele (OR = 0.594, 95% CI 0.388–0.909, *P* = 0.016). It was the same for the CC genotype compared to the AA genotype (OR = 0.286, 95% CI 0.091–0.899, *P* = 0.032), but the statistical significance was limited because there were only four patients and 16 controls with a CC genotype. Logistic regression analysis showed a significant association between IGFBP3 genotype and a decreased risk of acromegaly under the additive model (AA vs. AC vs. CC). After adjusting for age, sex, and smoking and drinking status, a multivariate logistic regression analysis revealed that the association remained significant under the additive model (OR 0.6, 95% CI 0.377–0.956) and the dominant model (AA vs. AC + CC) (OR 0.567, 95% CI 0.327–0.982) (Table 3).

**Table 3:** Association between the IGFBP3 rs2854744 genetic model and acromegaly risk

Genetic model	OR(95% CI)	*P* value	OR(95% CI)^a^	*P* value^a^
Additive	**0.594(0.388–0.909)**	**0.016**	**0.6(0.377–0.956)**	**0.032**
Dominant	0.604(0.358–1.019)	0.058	**0.567(0.327–0.982)**	**0.043**
Recessive	0.328(0.106–1.013)	0.053	0.378(0.115–1.237)	0.108

^a^Derived from logistic regression with adjustment for age, sex, and smoking and drinking status. Significant associations are marked in bold

### Analysis of rs2854744 and the risk of acromegaly in different subtypes

In the stratification analysis based on sex, we evaluated the association between the IGFBP3 genotype and acromegaly risk in female and male individuals. The χ^2^-test and risk estimation revealed statistically significant differences in the three genotypic distributions between female patients and controls (*P* = 0.011). Furthermore, there were significant associations between IGFBP3 genotype and decreased acromegaly risk under the additive (OR 0.385, 95% CI 0.206–0.72) and dominant (OR 0.364, 95% CI 0.168–0.785) models in females. However, there was not an association between IGFBP3 genotype and acromegaly risk in male individuals (Table 4).

**Table 4:** Stratification analysis for the rs2854744 genotype distribution and acromegaly risk by gender

Genotype^a^	Case [N(%)]	Control [N(%)]	OR (95% CI)	*P* value
	*n* = 102	*n* = 143		
Female				
AA	29(64.4)	29(39.7)		**0.011**
AC	15(33.3)	33(45.2)		
CC	1(2.2)	11(15.1)		
Additive			**0.385(0.206–0.72)**	**0.002**
Dominant			**0.364(0.168–0.785)**	**0.009**
Recessive			0.128(0.016–1.029)	0.054
Male				
AA	34 (61.8)	43(62.3)		0.917
AC	18(32.7)	21(30.4)		
CC	3(5.5)	5(7.2)		
Additive			0.963(0.527–1.762)	0.903
Dominant			1.021(0.492–2.12)	0.954
Recessive			0.738(0.169–3.236)	0.972

^a^Due to the missing values, genotypic data for 100 cases and 142 controls were available. Significant associations are marked in bold

Acromegaly can be classified into different subgroups according to its clinical and imaging characteristics. When comparing the patients of each subgroup with controls, we found a significant association between IGFBP3 genotype and a reduced acromegaly risk in the GH-secreting macroadenoma group (OR 0.557, 95% CI 0.347–0.893) and the monotherapy group (OR 0.512, 95% CI 0.316–0.828) under the additive model. Under the dominant model, there was also a significant association in the macroadenoma group (OR 0.524, 95% CI 0.295–0.932) and the monotherapy group (OR 0.545, 95% CI 0.308–0.964). However, IGFBP3 genotype was not associated with acromegaly risk in the other subgroups (Table 5).

**Table 5:** Association between IGFBP3 rs2854744 and acromegaly subtype risk

Subtype	Additive	Dominant	Recessive
	OR (95% CI)	OR (95% CI)	OR (95% CI)
Microadenoma	0.548(0.2–1.501)	0.643(0.201–2.06)	NA
Macroadenoma	**0.557(0.347–0.893)**	**0.524(0.295–0.932)**	0.432(0.139–1.34)
Combined therapy	0.907(0.455–1.808)	0.943(0.39–2.277)	0.75(0.161–3.5)
Monotherapy	**0.512(0.316–0.828)**	**0.545(0.308–0.964)**	**0.21(0.047–0.939)**

*NA* not applicable. Significant associations are marked in bold

### IGFBP3 level according to rs2854744 genotype

**Fig. 1:**
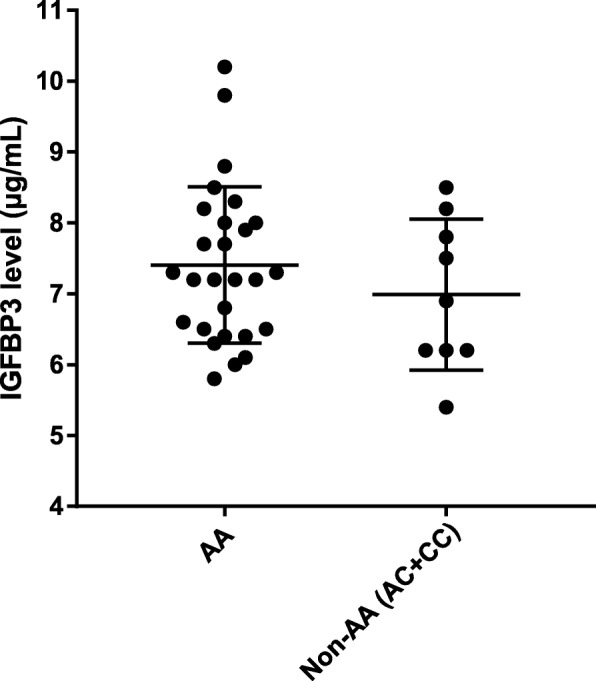
Serum IGFBP3 level before surgery according to genotype

The distribution of serum IGFBP3 levels as a function of rs2854744 genotype is shown in Fig. 1. A total of 36 blood samples from acromegaly patients before treatment were collected and tested for IGFBP3 levels; 27 patients had the AA genotype, 7 patients had the AC genotype, and only 2 patients had the CC genotype. Due to the limited number of subjects with the CC genotype, we combined the AC and CC groups. The serum IGFBP3 level was 7.404 ± 1.1 μg/mL in the AA group and 6.989 ± 1.065 μg/mL in the non-AA group. A higher IGFBP3 level was observed in patients with the AA genotype, but differences between the two groups were not statistically significant (*P* = 0.331).

## Discussion

In this acromegaly case-control study, we compared the rs2854744 genotype frequency in 102 acromegalic patients and 143 control group patients. In addition, we evaluated the association of the common polymorphism of IGFBP3 with the susceptibility to acromegaly, as well as with the susceptibility in subgroups of acromegaly. We found that IGFBP3 genetic variants were significantly correlated with an altered risk of acromegaly and that allele C of rs2854744 was strongly correlated with a decreased risk of acromegaly in the Han Chinese population.

IGFBP3 is the major transport protein for IGF-1, with IGFBP3 modulating the half-life and biological activities of IGF-1. Some cell culture studies have shown that IGFBP3 plays a vital role in cell survival or apoptosis in various microenvironments; meanwhile, several clinical studies have indicated that variations in IGFBP3 levels are associated with an altered risk for certain common cancers [14]. More than half of the changes in IGFBP3 levels were identified to be genetically determined by the gene polymorphism at the − 202 locus of IGFBP3 [9]. Therefore, we attempted to identify whether polymorphic variation at the − 202 site constitutes a risk factor for acromegaly, an important type of pituitary tumour disorder.

The rs2854744 polymorphism is situated 202 base pairs (bp) upstream of the transcription start site of IGFBP3 and has been reported to control promoter activity. The A allele has been proven to be correlated with higher promoter activity. The AA genotype was associated with higher IGFBP-3 circulating levels, which have been previously shown to be associated with a decreased risk of most cancers [15, 16]. This is because IGFBP3 commonly displays growth inhibition and pro-apoptosis properties, and IGFBP3 has been recognized as an inhibitor of IGF activity by blocking the binding of IGF to its receptor. In our study, we found that the AA genotype showed a tendency for an association with higher IGFBP3 serum levels, although the difference was not statistically significant (*P* = 0.331), which may be explained by the small sample size. At first, we hypothesized that the AA genotype at the − 202 site of the IGFBP-3 gene might be associated with a decreased risk of acromegaly. However, our results showed that the C allele was actually correlated with a decreased acromegaly risk. Using the A allele as a reference, the OR for the C allele was 0.594 (95% CI 0.388–0.909). The multivariate analysis showed that this association was still significant after adjusting for age, sex, and smoking and drinking status. This may be because IGFBP3 has been identified as being able to enhance the activity of IGF by protecting IGF from degradation under certain circumstances [17, 18]. In addition, IGFBP3 is capable of acting via an IGF-independent mechanism to promote cell growth and survival, such as by binding the 78 kDa glucose-regulated protein (GRP78), by inducing autography, and by promoting non-homologous end-joining (NHEJ) repair during genotoxic stress [14]. These results might explain why the acromegaly risk was decreased with the C allele in the current study.

Additionally, the varying allele frequencies among different ethnicities, geographies and populations may yield conflicting results regarding IGFBP3 polymorphisms and cancer risk. A large-scale urinary bladder cancer study based on a multiethnic population residing in Germany and Hungary demonstrated that the C allele (55%) was slightly more frequent than the A allele (45%) at the − 202 site of the IGFBP-3 gene [19], which was significantly different from our participants’ allele distributions. However, a cancer study involving Korean and Japanese [20] adults indicated that the A allele (65–80%) was appreciably more frequent than the C allele (20–35%) and that the C allele was correlated with a reduced non-small cell lung cancer risk [21]. The genotypic distribution and study results of the Asian population were similar to those of our population.

Furthermore, our results showed that the association between the C allele of rs2854744 and a decreased risk of acromegaly was more significant in females, those with large tumours and those treated with monotherapy. IGFBP3 levels have been reported to be correlated with the stage and prognosis of cancer [6, 22, 23]. Several studies have found that some SNPs have different effects on cancer susceptibility between genders [24, 25]. We analysed genetic polymorphism and acromegaly risk in different subgroups. The results of our study were in some accordance with the results of the previously mentioned studies.

Our study had a few advantages. First, this was one of the first acromegaly case-control studies examining the relationship between the IGFBP3 -202A/C polymorphism and acromegaly risk. Second, we conducted an analysis of subgroups according to sex, treatment method and adenoma size, since pituitary adenomas possess diverse clinical manifestations, and the IGFBP3 gene may be associated with a specific subtype.

However, several limitations existed in the present study. First, we only selected acromegaly inpatients confirmed by postoperative pathology from Beijing Tiantan Hospital, which might have led to a selection bias. Second, as the groups with a CC genotype had fewer participants, comparisons with the AA group had limited statistical significance. Third, we did not perform functional research to confirm our findings.

## Conclusion

In summary, this was the first study that explored the relationship between the IGFBP3 -202A/C polymorphism and a predisposition to acromegaly in a Chinese population. Our findings revealed that IGFBP3 genetic variants are associated with acromegaly risk. The C allele of rs2854744 was associated with a decreased risk of acromegaly. As mentioned above, more studies that include functional research and that focus on other populations are needed to confirm and further characterize the association.

## Abbreviations

bp: Base pair
CI: Confidence interval
EDTA: Ethylene diamine tetraacetic acid
GH: Growth hormone
GRP78: 78 kDa Glucose-regulated protein
HWE: Hardy-Weinberg equilibrium
IGF-1: Insulin-like growth factor-1
IGFBP3: Insulin-like growth factor binding protein-3
IGFBPs: IGF-specific binding proteins
MAF: Minor allele frequency
MRI: Magnetic resonance imaging
NHEJ: Non-homologous end-joining
OR: Odds ratio
PCR: Polymerase chain reaction
SD: Standard deviation
SNP: Single nucleotide polymorphism
